# Implementation of patient pagers in radiation oncology waiting rooms for patient privacy and satisfaction

**DOI:** 10.1186/s13104-018-3164-5

**Published:** 2018-01-22

**Authors:** Tasneem Kaleem, Daniel Miller, Mark R. Waddle, Maresciel Yanez, Bonita Gianforti, Steven Buskirk

**Affiliations:** Department of Radiation Oncology, Mayo Clinic Florida, 4500 San Pablo St, Jacksonville, FL 32224 USA

**Keywords:** Radiation, Waiting room, Pager, Privacy

## Abstract

**Objective:**

In order to improve privacy, quality, and coordination of care, a patient pager system was introduced to notify patients of daily treatment in the Department of Radiation Oncology. One hundred patients undergoing daily radiation therapy prospectively participated in a six-question survey addressing the paging service, privacy prior to pager use, and demographics. Twelve radiation therapists also participated in a survey addressing privacy and workflow.

**Results:**

Survey results from all patient participants revealed that convenience, privacy, ease of use, desire for use for consults and return visits were highly rated as very good to excellent. The top three categories were “ease of use,” “convenience” and “privacy.” Nineteen patients had the experience of our waiting room prior to introduction of the patient pagers and highly rated “privacy,” “efficiency,” and “satisfaction.” Twelve radiation therapists participated and rated workflow related categories fair to good. Only patient privacy was rated as very good to excellent. Thus, patients and staff highly rated the paging system for privacy protection and satisfaction. However, it did not change overall workflow. Our study shows clinics should prioritize privacy in the waiting room to address the emotional needs of patients and improve satisfaction.

## Introduction

Logistically, radiation treatment is unlike many other medical interventions. Most other treatments are a 1 day procedure requiring a one-time visit to the clinic or hospital. In contrast, radiation is usually a daily affair, requiring the patient to become a frequent flier to the clinic for up to 8 weeks. Thus, the waiting room becomes a familiar setting. Despite its familiarity, privacy continues to be a valued virtue among patients in the outpatient setting. A recent survey of patient’s perspective at an outpatient clinic showed that confidentiality at the outpatient clinic was a major concern [1]. Thus, patient privacy is an important component of patient satisfaction.

Moreover, with the growing population of oncologic patients, quality and coordination of care becomes a priority. Efficient treatment of a large number of daily patients requires organization and time management tools. The normal process of daily radiation treatments is as follows:

- Patient checks into front desk.
- Therapists watches EMR for “checked in” patient on schedule.
- Therapist leaves treatment bay and goes to the waiting room.
- Therapist then verbally recalls patient in the waiting room.
- Therapist accompanies patient to changing room.
- Patient then goes to treatment bay as Therapist wait for patient to change into a gown and then accompany to treatment machine. Treatment occurs.

This process can take time out of the daily workflow for radiation therapist’s time, and is repeated up to 30 times per day. In an effort to address both privacy and service improvement, we implemented patient paging system that allowed removal of the verbal recall and more autonomy to the patient to go the changing room after pager notification. This process allows the therapist to continue preparation for the next treatment without interruption. Utilization of a paging system reduces the daily process to four steps since recall is performed electronically.

The concept was inspired by the restaurant industry, where beepers are given to customers awaiting seating. Within a medical environment, waiting room patient pagers have been previously studied in the perioperative setting. Paging devices have shown to be helpful for individuals awaiting news about family members’ surgeries. Pagers were an adequate tool for intraoperative communication and reduce anxiety [2, 3]. However, there has been no prior report of pager use for patients in radiation oncology waiting room settings. The purpose of this study was to identify and evaluate waiting room patient pagers as a method to improve patient privacy and coordination of care in the daily radiation treatment setting.

## Main text

### Methods

A total of 100 on treatment radiation oncology patients were enrolled in the study. The study was twofold, with 42 patients participating in surveys in June 2016, and a subsequent evaluation of 58 patients 1 year later in June 2017. Sample size was determined in a temporal manner, thus all patients eligible and willing to participate in the study within the month of June 2016 and June 2017 accrued. One month time period was chosen to ensure capturing patients on shorter as well as longer radiation regimens without risking duplication. The two time points allowed data to be gathered early during the implementation process as well as 1 year later after more seasoned use. Any patient undergoing at least one treatment of radiation at our institution was eligible, with no restriction on age, gender, primary site, radiation technique, or treatment intent. Exclusion criteria include patients unable to answer survey due to disability or performance status, previous survey participation or refusal to participate. Six radiation therapists were involved with linac based treatment, thus a total of 12 therapists over the course of the two study time points. The setting is a busy radiation oncology outpatient waiting room.

Apollo pagers with light and vibration notification were numbered and added to mini clipboards with instructions and handed to the patients at time of check in. Front desk staff was responsible for patient orientation to the paging system on Day 1 of treatment, checking in patient, inputting pager number into scheduling system, handing the pager to the patient, as well as collecting and sanitizing pager after it is returned prior to treatment. Once patient is checked in and treatment bay is ready, the radiation therapist would activate pager which would alert patient to go to changing room and report to treatment machine. Activation is performed through the hospital phone system by dialing the pager number.

**Fig. 1:**
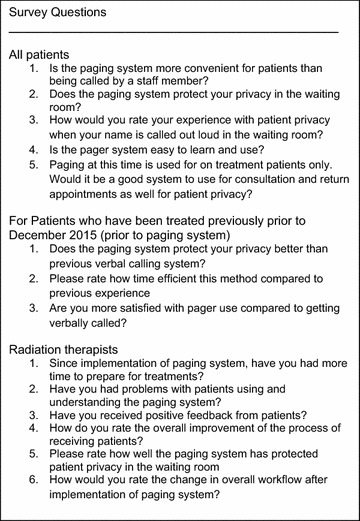
Survey questions. Participants rated their experience on a scale of 1–5 (1 being poor, 5 being excellent)

Patients participating in the study answered a six-question survey addressing various aspects of the paging service, privacy prior to pager use, and demographics (Fig. 1). Demographics included gender, age range, treatment intent and number of treatments. Twelve radiation therapists also participated in a 7-question survey addressing privacy and workflow. Participants rated their experience on a scale of 1–5 (1 being poor, 5 being excellent). Surveys were collected and retrospectively reviewed.

### Results

From June 2016 to June 2017 a total of 100 patients undergoing radiation therapy at the department of radiation oncology at Mayo Clinic Florida participated in the survey. Forty two patients completed the survey in 2016 and 58 patients completed the survey in 2017. Demographic information was completed by 55 patients. The majority of patients who answered were female, about 55%, and greater than 90% of this cohort was over 50 years old. Only 1 patient had less than 5 treatments planned, 12 patients had 10–20 treatments, 30 patients had 20–30 treatments, and 12 patients had more than 30 treatments.

**Fig. 2:**
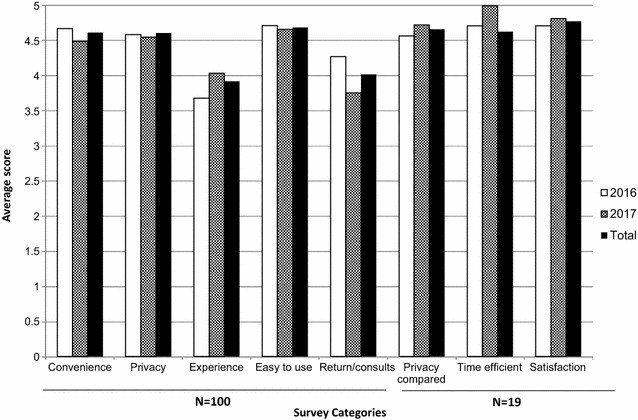
Patient Survey results. There was no difference between average results between the two time points. All categories were very good to excellent except for “experience” and “pager use for returns and consults” which were good to very good. 19 patients experienced the waiting room prior to pager implantation and rated all categories in comparison to prior protocol very good to excellent

The first sets of questions were answered by all 100 participants. The top category was “ease of use” (4.69), followed by “convenience” (4.62), “privacy” (4.61), “possible use for Returns/consults” (4.02), and “overall experience” (3.92). All of the categories were rated very good to excellent except for “overall experience”, which was rated good to very good (Fig. 2). There was no difference in averages for each category for the different time points.

**Fig. 3:**
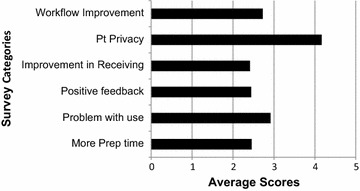
Staff Survey results. All categories were very fair to good except for “patient (Pt) privacy” which was rated very good to excellent

A cohort of 19 patients had the experience of our waiting room prior to introduction of the patient pagers. When asked to compare the use of pagers in the current waiting room procedure compared to prior protocol, patients highly rated “Privacy” (4.67), “Efficiency” (4.63) and “Satisfaction” (4.73) with pagers. All categories were rated Very Good to Excellent (Fig. 3).

Radiation therapists pooled together from both 2016 and 2017 time points participated in the survey for staff perspective. A total of 12 therapists, 6 from each time point, answered the survey. The highest rated category was “patient privacy” (4.17). The rest of the categories were in the fair to good range, which included “Patient Problems with use” (2.91), “Improvement in workflow” (2.72), “More preparation time” (2.45), “Positive Feedback from patients” (2.44), and “Improvement in process” (2.42) (Fig. 3).

### Discussion

To our knowledge, this is the first study to correlate waiting room paging device with patient privacy and satisfaction. Both patients and staff at our institution recognized improved privacy with pager use. Moreover, this is the first to evaluate introduction of technical device for patients within waiting rooms of radiation oncology.

Patient pagers have been previously evaluated as devices to aide emotional needs of patients and satisfaction. Several studies have evaluated pagers in the perioperative and intraoperative settings. The most recent study assessed the use of pagers intraoperatively as an effective means of communication. Pagers were easy to use for families, doctors and nurses. Overall, pager use showed a 30% improvement in satisfaction [4]. Other studies evaluated the impact of paddle pagers on family anxiety during the intraoperative period in a quasi-experimental fashion. Family members were either given a pager or verbal communication. Verbal family communication had a greater significant difference in anxiety scores preoperative vs postoperative [2].

Beyond the operative setting, electronic pagers have also been used in outpatient based chemotherapy waiting rooms. Chemotherapy is similar to radiation in terms of requiring multiple scheduled visits over long period of time. Introduction of pagers in the waiting room provide patients a greater sense of control over time while waiting. Staff also reported a benefit in terms of facility to recall patients, work intensity and less aggression [5].

In our study, patients highly rated the convenience, privacy, and ease of system. This was not different when comparing early in the implementation versus 1 year later after the staff was more accustomed to the process. This lack of difference is most likely due to the straightforward manner and simplicity of the pager. Patients who experienced the waiting room protocol prior to pager implementation highly rated the new system in terms of satisfaction, privacy protection, and efficiency. This cohort is able to subjectively compare waiting room environments and is a valuable asset to the study. They have experienced the same waiting room before and after implementation of pagers. These results show that there is an overall improvement with the use of pagers.

Staff highly rated patient privacy, however did not find significant advantage in terms of work flow. A number of staff reported many patients not answering pagers in a timely fashion or operating pager efficiently. There have been occasions where the incorrect pager number was inputted, and subsequently wasting time to verbally call the patient. Front desk staff mentioned they are contacted at least three times per day regarding the status of a patient in the waiting room after paged. Furthermore, some pagers have also required replacement due to malfunction or breakage.

Given these issues with workflow, we have developed a focus group to address patient workflow with pagers. Considerations include a direct paging system rather than calling pager through the phone system. Other possibilities including upgrading pagers or considering a camera in the waiting room to be viewed by the therapists. This would allow direct visualization of the patients.

Patient pagers first and foremost address the emotional need of patient privacy and thus translate to satisfaction. This study shows patients highly value privacy and convenience. Pagers are a simple tool which can provide communication without language or disability barriers. Despite the fact that certain technical aspects may have hindered improvement in workflow, waiting room paging devices should be considered by other radiation oncology departments to help address patient needs.

## Limitations

We acknowledge the limitations of our study, including low sample size and single institution design. Our study does provide new information, however in the context of a relative paucity of outcome metrics. Endpoints were limited to patient satisfaction and workflow. Moreover, only a portion of participants were compliant with demographic information, which was patient biased rather than obtained through chart review. Furthermore, due to the implementation of pagers and subsequent data collection, we were not able to obtain adequate data of satisfaction prior to introduction of pagers. Only a small number of patients were recurrent and eligible to evaluate waiting room procedure prior to pager implementation.

## Abbreviations

Pt: patient
Ret: return visit
